# TRAF3 as a Multifaceted Regulator of B Lymphocyte Survival and Activation

**DOI:** 10.3389/fimmu.2018.02161

**Published:** 2018-09-24

**Authors:** Gail A. Bishop, Laura L. Stunz, Bruce S. Hostager

**Affiliations:** ^1^Department of Microbiology & Immunology, University of Iowa, Iowa City, IA, United States; ^2^Department of Internal Medicine, University of Iowa, Iowa City, IA, United States; ^3^Iowa City VA Health Care System, Iowa City, Iowa City, IA, United States

**Keywords:** TRAF, B cell, signal transduction, cytokine, toll-like receptor, TNF receptors, cancer

## Abstract

The adaptor protein TNF receptor-associated factor 3 (TRAF3) serves as a powerful negative regulator in multiple aspects of B cell biology. Early *in vitro* studies in transformed cell lines suggested the potential of TRAF3 to inhibit signaling by its first identified binding receptor, CD40. However, because the canonical TRAF3 binding site on many receptors also mediates binding of other TRAFs, and whole-mouse TRAF3 deficiency is neonatally lethal, an accurate understanding of TRAF3's specific functions was delayed until conditional TRAF3-deficient mice were produced. Studies of B cell-specific TRAF3-deficient mice, complemented by investigations in normal and malignant mouse and human B cells, reveal that TRAF3 has powerful regulatory roles that are unique to this TRAF, as well as functions context-specific to the B cell. This review summarizes the current state of knowledge of these roles and functions. These include inhibition of signaling by plasma membrane receptors, negative regulation of intracellular receptors, and restraint of cytoplasmic NF- κB pathways. TRAF3 is also now known to function as a resident nuclear protein, and to impact B cell metabolism. Through these and additional mechanisms TRAF3 exerts powerful restraint upon B cell survival and activation. It is thus perhaps not surprising that TRAF3 has been revealed as an important tumor suppressor in B cells. The many and varied functions of TRAF3 in B cells, and new directions to pursue in future studies, are summarized and discussed here.

## Introduction

We will begin with a brief summary of work leading up to the current understanding of the multiple important roles played by TRAF3 in B lymphocytes; the reader is referred to previous reviews for information on roles of TRAF3 in other cell types (1–6). TRAF3 was discovered as the first protein demonstrated to associate with the cytoplasmic domain of the tumor necrosis factor receptor (TNFR) superfamily member CD40 (7, 8). TRAF3 also binds the C-terminal cytoplasmic domain of the Epstein Barr virus (EBV)-encoded CD40 mimic, Latent Membrane Protein 1 (LMP1) (9). It was inevitable that this newly-identified signaling protein would be experimentally deleted from the mouse germline, the only technology widely available at the time to create “knockout” mice. As with many broadly-expressed signaling proteins, this whole-mouse germline *Traf3* deletion resulted in early lethality (10), and thus could provide only limited hints of TRAF3 protein function, particularly for specific mature cell types. Interestingly, however, this initial report suggested regulation of T-dependent antibody production by TRAF3, a role that was confirmed much later, when T cell-specific TRAF3-deficient mice were made and analyzed (11).

As CD40 was the first identified TRAF3 binding receptor, studies followed examining the role of TRAF3 in CD40 signaling to B cells. Several groups obtained evidence that TRAF3 plays an inhibitory role in both CD40 signaling (12–14), as well as synergistic signaling mediated by cooperation between CD40 and the B cell antigen receptor (BCR) (15, 16). TRAF3 also inhibits signaling to B cells by the BAFF receptor (BAFFR) (17).

Pinning down TRAF3's role precisely was initially prevented by the highly overlapping nature of the major binding site on CD40 (and many other TRAF-binding receptors) for TRAFs 1, 2, 3, and 5 (PXQXT) (18). Thus, the available approaches of mutating the receptor's binding site, and/or mutating the TRAF3 molecule to prevent receptor binding (creating a “dominant negative” TRAF3) could provide important information, but ultimately could not lead to unambiguously interpretable data, because both strategies impact the nature and stoichiometry of binding of other types of TRAFs, in addition to TRAF3. The stoichiometric abnormalities were particularly acute in model systems using exogenous overexpression of TRAF molecules and receptors, such as 293 epithelial cells. For example, a point mutation in the major PXQXT CD40 cytoplasmic domain motif obviates binding of both TRAFs 2 and 3 in artificial systems (18), but when this mutant CD40 molecule is expressed at approximately normal levels in B cells, it binds TRAF3 indistinguishably from WT CD40 (15).

Prior to wide availability of the Cre-Lox system for conditional deletion of specific genes in B cells (19), the challenge of the overlapping TRAF binding site was addressed using modification of gene targeting by homologous recombination, tailored to use in somatic cell lines, which allows complete and specific removal of single types of TRAF molecules (20). When this approach was applied to TRAF3 in B cell lines, a surprising result was obtained. In B cells inducibly expressing transfected LMP1 plus endogenous CD40, removal of TRAF3 enhances CD40 signaling—consistent with earlier reports—but greatly inhibits the typically amplified signaling induced by LMP1 in the same B cells (21). It was subsequently revealed that CD40 and LMP1 bind TRAF3 in distinct ways, contributing to this striking difference (22, 23). Thus, TRAF3 can play distinct roles in regulating signaling to the same cell by different receptors.

Following discovery of the mitogen-activated protein kinase kinase kinase (MAP3K) called NF- κB inducing kinase (NIK), and its important role in activation of the non-canonical/NF- κB2 pathway by TNFR superfamily members (24, 25), it was shown that activation of this pathway by CD40 also involves NIK (26). While this finding was initially made in 293 epithelial cell line overexpression models, it was subsequently confirmed in B lymphocytes (27). Importantly, TRAF3 was revealed to be a master regulator of NIK stability in multiple cell types, including B cells (28).

**Figure 1:**
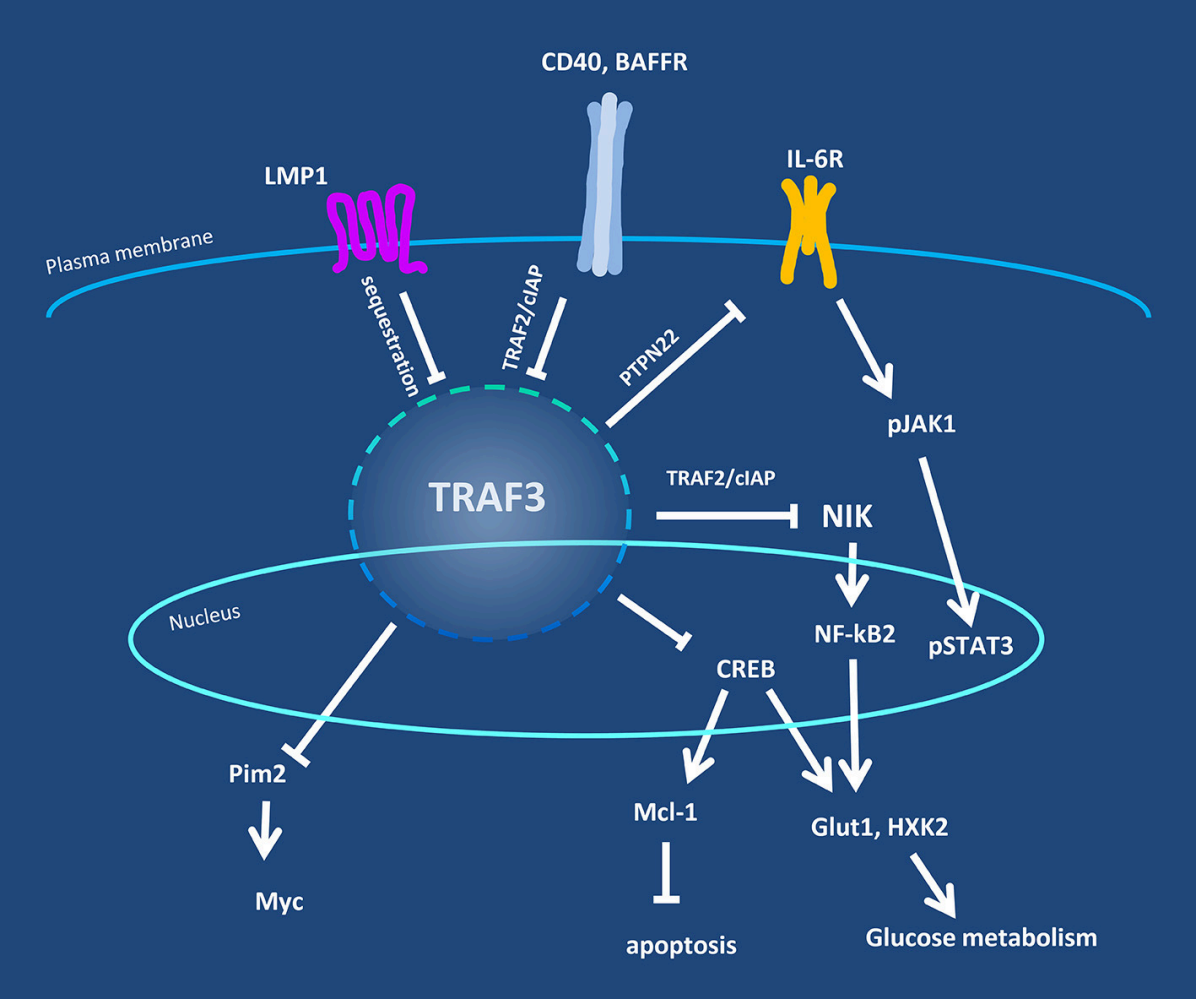
Overview of TRAF3 regulatory pathways in B lymphocytes. Levels of TRAF3 protein and/or its availability in B cells are regulated by cell surface-expressed receptors, exemplified by CD40, BAFFR, and the viral protein LMP1. TRAF3 is in turn responsible for regulation of the activity of additional signaling proteins in the cytoplasm and nucleus, including NIK, Pim2, and CREB. Negative regulatory partners or mechanisms are indicated by the crossbar pointers.

Building upon all these earlier studies, the best information to date on the multiple roles played by TRAF3 in B cells has derived from strains of mice lacking *Traf3* expression specifically in B cells. The first two strains of this type were reported in 2007 (29) and 2008 (30); they revealed a newly appreciated critical role for TRAF3 in restraining B cell homeostatic survival. Consistent with the previously reported role for TRAF3 in reducing NIK stability, TRAF3-deficient primary B cells display constitutive p100 processing and nuclear p52 and Rel B (29). However, subsequent mice made TRAF3-deficient in T lymphocytes, dendritic cells, or macrophages all also display constitutive NF- κB2 activation in the TRAF3-deficient cells, but only TRAF3-deficient B cells display enhanced survival (11, 31, 32). Thus, in addition to receptor-specific roles, TRAF3 has cell-type specific functions, and exerts particularly unique and critical regulation of signaling pathways in B cells. The current state of knowledge of these functions and pathways will be discussed in the following sections, and are summarized in Figure 1.

## Interactions between TRAF3, the B cell antigen receptor (BCR) and TNFR superfamily (TNFRSF) receptors

Numerous TNFRSF members expressed by B lymphocytes interact (or potentially interact) with TRAF3. These receptors include CD27, CD30, BCMA, CD40, BAFFR, TACI, TNFR2, and 4-1BB.

### CD40 and CD40^+^BCR

CD40 is a somewhat unusual member of the TNFRSF in that it activates both the canonical/NF-κB1 and non-canonical/NF-κB2 pathways. TRAF proteins play important roles in regulating signaling pathways activated by CD40 (33). Following engagement of CD40 by CD154 (CD40L) or agonistic antibody, the cytoplasmic domain of CD40 binds TRAF1, TRAF2, TRAF3, TRAF5, and TRAF6 (34). TRAF3 negatively regulates CD40 signaling in B cells (12). Studies of CD40 and TRAF mutants in B cell lines indicate that TRAF3 also has a negative role in regulating synergy between CD40 and the BCR in activation of antibody and cytokine secretion (15). Transformed B cell lines deficient in TRAF3 exhibit markedly enhanced CD40-mediated activation of c-Jun kinase (JNK) and antibody secretion (21), although CD40-mediated signals (including the activation of JNK, p38, ERK, NF- κB1, and NF- κB2), in TRAF3-deficient primary B cells appear only modestly enhanced (29, 30).

### TNFR2 (CD120b)

TNFR2 contributes to the activation of antibody secretion by B cells, mediating autocrine stimulation of B cells by CD40-induced TNF (35). TRAF3 is recruited to this receptor (36), and one role in its regulation of signaling by TNFR2 in B cells may be in the activation of JNK. TNFR2 activates both the canonical and non-canonical NF- κB pathways in B cell lines, although activation of the canonical pathway appears weak (36). TRAF2 contributes to NF- κB signaling activated by TNFR2, but TRAF3 may contribute as well, as B cells deficient in TRAF2 exhibit reduced, but not absent, TNFR2-mediated NF- κB2 activation (36). Further discussion is provided in the section on TRAF3 and B cell cytokine receptors, below.

### 4-1BB (CD137)

While signaling by 4-1BB (ligand = CD137L, 4-1BBL) has been evaluated in T cells, the effects of 4-1BB signaling in B cells are less well-characterized (reviewed in (37). In mammals, a small population of 4-1BB-expressing B cells expands with age, and in collaboration with cytotoxic T cells may be important in slowing tumor growth (38). 4-1BB, whose expression can be upregulated in B cells by various stimuli, is capable of enhancing B cell proliferation and TNF production [reviewed in (39)]. The cytoplasmic domain of 4-1BB binds TRAF1, TRAF2, and TRAF3 (37). However, the biological roles of TRAF3 in 4-1BB signaling in B cells remain unclear.

### CD27

In humans, CD27 is a marker for memory B cells and promotes differentiation of B cells to plasma cells; its ligand is CD70 (40). In epithelial cells, CD27 has been shown to bind TRAF3 (41, 42), where it can potentially inhibit the activation of NF-κB mediated by TRAFs 2 and/or 5 (42). As is the case for 4-1BB, how TRAF3 regulates CD27 function in B cells remains undefined.

### CD30

Small numbers of human tonsillar mononuclear cells are CD30^+^ B cells. These cells appear to be a subpopulation of B cells that develop during normal germinal center reactions in lymphoid tissue, and share some transcriptional patterns with Hodgkin lymphoma cells (43). In non-B cell systems, CD30 can bind TRAFs 1, 2, and 3 (44, 45). In mouse T cells, engagement of CD30 by its ligand CD30L/CD153 can activate p38, JNK, and NF-κB. Interestingly, a dominant-negative TRAF2 could inhibit CD30-induced p38 and JNK activity, but not NF-κB activation (46). The role of TRAF3 in CD30 signaling in B cells is currently unclear.

### Receptors for B cell activating factor (BAFF) and a proliferation-inducing ligand (APRIL)

TRAF3 plays a major role in signaling by at least one of the receptors for BAFF and APRIL. The cytoplasmic domain of BAFF receptor (BAFFR/CD268) interacts with at least three TRAF proteins, TRAF2, TRAF3, and TRAF6 (47). Engagement of TRAF2/3 by BAFFR leads to proteasomal degradation of TRAF3 and activation of the non-canonical NF- κB2 pathway (see section on NF-κB activation below). Targeted disruption of TRAF3 expression in B cells mimics treatment of normal B cells with BAFF, resulting in enhanced BAFF-independent B cell survival (29). However, experiments with mutant TRAF3 and mutant BAFFR molecules reveal that TRAF3 degradation may be neither necessary nor sufficient for NF-κB2 activation (48). BAFFR also activates the canonical NF-κB1 pathway in B cells, and this signaling event is mediated not by TRAF3, but by TRAF6 (47). Signaling by BAFFR also activates the kinases Syk, phosphatidyl inositol 3-kinase (PI3K), and ERK in B cells (49, 50). The activation of PI3K appears to be TRAF3-independent (50).

The TNFRSF receptors, transmembrane and CAML interactor (TACI) and B cell maturation antigen (BCMA), are also docking sites for BAFF, and a second TNF family member, APRIL. TACI is expressed by activated B cells and plasma cells (51), and interacts with TRAF3 (37). TACI-deficient mice display a marked increase in overall B cell numbers and increased antibody production, indicating an important role in B cell homeostasis (52–54). The role of TACI is complex, however, and not limited to negative regulation of B cells. In humans, *TACI* gene defects are detected in ~8% of all cases of common-variable immune deficiency (51). TRAF3 inhibits the NF- κB2 pathway activated by TACI in a human kidney epithelial cell line (14). The role of TRAF3 in B cell TACI signaling remains to be described. Function of the BCMA receptor is critical for the long-term survival of antibody-secreting plasma cells (reviewed in (55, 56). While BCMA can bind TRAF3 in non-B cell over-expression systems (57, 58), the role of TRAF3 in BCMA signaling in B cells is not yet defined.

## TRAF3 and TLRs in B cells

Toll-like receptors (TLRs) comprise a group of 13 transmembrane receptors in mammals, expressed on innate immune cells as well as B and T cells [reviewed in (59–61)]. These molecules initiate signaling cascades in response to binding molecules containing certain pathogen or disease associated molecular patterns, thereby regulating the production of type I interferons (IFNs) and other cytokines (60, 62, 63). TRAF6 was initially thought to be the single TRAF involved in TLR signaling (64), but it later became clear that TRAF3 interacts with the TLR pathway adapter proteins MyD88 and TRIF, and regulates TLR signaling using alternative pathways for each (60, 62, 65).

A key finding in the literature showing the importance of TRAF3 to TLR signaling is a case report describing a patient with a history of pediatric *Herpes simplex* viral encephalitis (66). Genetic analysis showed a single allele amino acid substitution in *TRAF3* that exerted a dominant negative effect by mediating a decrease in cellular levels of wild-type TRAF3 protein. The patient's myeloid cells were poor producers of type I IFNs when stimulated *in vitro* with the TLR ligands poly(I:C), LPS or R848, highlighting the importance of TRAF3 in these responses (66).

In studies utilizing mice with *Traf3* deleted specifically in either dendritic cells (DC) or B cells, it was observed that in the absence of TRAF3, DCs respond to signaling through TLRs 3,4,7 and 8 with either no change or decreases in IL6, TNF, and IL-10 (67). TRAF3-deficient DC exhibit a reduced activation-induced type I IFN response (65, 68). In contrast, TRAF3-negative B cells show increased production of TNFα, IL-6, and IL-10 (67). Thus, there is cell-type specific regulation of TLR-driven cytokine production by TRAF3. This difference extends to the IFN pathway, with TRAF3-deficient B cells producing more phosphorylated IFN-response factor 3 (IRF3) and IFNγ-induced protein 10 in response to stimulation through TLRs 3, 4, 7, and 9 (67). Furthermore, B cells lacking TRAF3 also show increased levels of activation induced deaminase and production of isotype-switched immunoglobulins in response to TLR stimulation (67).

In mouse macrophages, *Traf3* mRNA and protein expression is increased as a consequence of TLR2 stimulation (69). Increased TRAF3 levels are critical to enhanced IRF3 activation and *IFN*β gene induction in response to subsequent signals through TLRs 3 or 4 (69). Conversely, TRAF3 levels can be depleted in B cells after engagement of CD40 and BAFFR, via ubiquitination and degradation of TRAF3 (reviewed in (33). Deubiquitinases can interrupt the process of TRAF3 degradation (70), as well as disrupt the function of signaling complexes containing TRAF3 (6). Thus, regulation of TRAF3 protein levels and modifications can act as a rheostat, affecting the outcome of signaling to B cells and other cell types via TLRs; the direction of the regulation seen appears to be both receptor and cell-type-specific.

## TRAF3 and B cell cytokine receptors

As discussed above, TRAF3 serves to regulate TLR signaling to B cells in various ways, impacting a number of downstream TLR-induced events, including cytokine production. However, the involvement of TRAF3 in regulating signals induced by the receptors for such cytokines is much less understood, particularly for the cell of focus in this review, the B lymphocyte.

The first known members of the TNFR superfamily are receptors for the cytokine TNF themselves—TNFR1/CD120a and TNFR2/CD120b. While CD120a is expressed in modest to undetectable levels on B cells, CD120b is robustly expressed (35). The roles played by TRAF2 in signaling to various cells by CD120b is well-documented [reviewed in (71)], including signals to B cells, in which TNFR2 plays important roles in Ig production (35, 36). Because TRAF2 binds CD120b, and TRAF2 often forms heterodimers with TRAF3, it was predicted that TRAF3 is also a CD120b-associated protein (71). This prediction was subsequently confirmed for HEK293 epithelial cells transfected with plasmids encoding CD120b and TRAF3; in this system, TRAF3 inhibits NF-κB and JNK activation induced by exogenous over-expression of both CD120b and TRAF2 (72). In B lymphocytes, endogenously-expressed CD120b also binds TRAF3, and recruits this adapter to membrane lipid rafts (36). Similar to CD40 signaling to B cells, CD120b engagement induces both TRAF2 and TRAF3 degradation (36). However, how B cell TRAF3 regulates CD120b signaling in lymphocytes remains to be discovered, an important knowledge gap.

The receptor for the cytokine IL-17 binds TRAF3 when both the receptor and TRAF3 are exogenously overexpressed in the fibroblast cell line HeLa or the epithelial cell line HEK293 (73). Using the same model systems, it was subsequently shown that TRAF3 competes for IL-17 receptor binding with the pro-inflammatory kinase nuclear Dbf2-related kinase (74). Whether these associations can be confirmed for endogenous levels of these proteins in immune cell types will be of great interest for future investigation.

Conditional *Traf3*-deficient mice, produced by Cre-Lox technology (29), revealed two additional cytokine receptors that are regulated by TRAF3 in lymphocytes. Mice lacking TRAF3 in T cells have a 2-3-fold increase in natural T regulatory cells (Treg), attributable to enhanced IL-2 receptor (IL-2R) signaling to pre-Treg (75). In WT T cells, TRAF3 mediates recruitment of the phosphatase T cell protein tyrosine phosphatase (TCPTP, also known as PTPN2) to the IL-2R. TCPTP de-phosphorylates the IL-2R-associated Janus kinase (Jak) 2 and the transcription factor signal transducer and activator of transcription (Stat) 5 (75). Thus, in TRAF3-deficient T cells, there is enhanced Jak2 and Stat5 phosphorylation, and amplified signaling through the IL-2R (75). It is not yet known whether IL-2R signaling is altered by TRAF3 in B cells; given the striking cell-type specificity of TRAF3-mediated regulation, this is an interesting knowledge gap to be addressed.

The IL-6R is the cytokine receptor for which we have the most detailed understanding of the regulatory role for TRAF3 in B cells to date. Investigation of this relationship was prompted by the observation that B-*Traf3*^−/−^ mice, in addition to their phenotype of increased homeostatic survival of all B cells (29, 30), display a 2-3-fold increase in CD138^+^ plasma cells (76). This increase disappears in B-*Traf3*^−/−^ mice bred to IL-6^−/−^ mice, although B-*Traf3*^−/−^ mice have no increase in IL-6R levels, nor of serum IL-6 (76). These results implicated IL-6R signaling as responsible for the enlarged plasma cell compartment. Upon investigation it was revealed that B cells lacking TRAF3, similar to the situation with the IL-2R in TRAF3-deficient T cells described above, show elevated IL-6-induced phosphorylation of Jak1 and Stat3, the signaling molecule pair equivalent to that of Jak2 and Stat5 for the IL-2R. In both cases, normal human peripheral blood T or B cells transduced with siRNA targeting human TRAF3 also display increased lymphokine-mediated Stat phosphorylation (75, 76). As in T cells, the mechanistic explanation for this phenotype is that TRAF3 is recruited to the IL-6R upon cytokine binding, to which it recruits a phosphatase—in the case of B cell IL-6R, this is PTPN22 (76). Consistent with these results, *Ptpn22*^−/−^ mice also display an increased plasma cell compartment and elevated pStat3 following IL-6 signaling (76). It will be exciting to determine how widespread this novel role for TRAF3 in recruitment of phosphatases is, and its specificity in regards to both cell and receptor type.

## Regulation of NF- κB cytoplasmic pathways

Most, if not all members of the TNFRSF are capable of activating NF- κB. In general, family members that interact with TRAF2 or TRAF6 induce the canonical NF-κB1 pathway through activation of inhibitor of kappa B (I κB) kinase β (IKKβ) (77). IKKβ is responsible for phosphorylating NF-κB inhibitory IκB proteins, flagging them for poly-ubiquitination and proteasomal degradation. Destruction of these inhibitors releases components of the canonical pathway, such as p50, to transit to the nucleus and initiate transcription of various genes [reviewed in (78)]. TNFRSF members such as CD40 and BAFFR that bind TRAF3, also often activate the non-canonical NF-κB2 pathway [reviewed in (79)]. Interestingly, in this pathway, TRAF3 serves a negative regulatory function crucial for normal B cell homeostasis [reviewed in (33)]. In the context of CD40 and BAFFR signaling, and potentially in signaling by other receptors that interact with TRAF3, the recruitment of TRAF3 to the receptor disrupts TRAF3's inhibitory activity in the cytoplasm, allowing activation of the NF-κB2 pathway. In unstimulated cells, TRAF3 forms a complex with TRAF2 and cellular inhibitors of apoptosis (cIAPs) 1 and/or 2 [reviewed in (80)]. This complex regulates the MAP3K NF-κB inducing kinase (NIK), an enzyme that phosphorylates and activates IKKα, which in turn is responsible for mediating the phosphorylation of p100, a precursor component of the NF-κB2 pathway. In unstimulated cells, the TRAF3/cIAP1/2 complex induces the post-translational modification of NIK with K48-linked poly-ubiquitin, targeting it for proteasomal degradation. This prevents NIK from contributing to the phosphorylation of p100 by activating IKKα, which would otherwise lead to p100 processing into active p52, an important component of the NF-κB2 pathway. Engagement of CD40, or other TNFRSF members that interact with TRAF3, can direct the ubiquitination activity of cIAP1/2 on to TRAF3 itself, resulting in its ubiquitination and degradation. The decrease in cytoplasmic TRAF3 leads to accumulation of NIK in the cytosol, which is then able to process p100, leading to translocation of p52 (often as a heterodimer with RelB) into the nucleus [reviewed in (80)]. The degradation of TRAF3 may not be strictly required for this to occur (48); its redirection away from NIK may suffice. TRAF3 may also help regulate the canonical NF-κB1 pathway, by interfering with the binding of other TRAFs to the cytoplasmic domains of stimulatory receptors such as CD40 (30). In addition to its regulation of NF-κB2, NIK appears to regulate NF-κB1 through its activation of the IKK complex (81). The regulation of NIK levels by TRAF3 may therefore also modulate NIK-mediated NF-κB1 activity.

## B cell TRAF3 and nuclear functions

TRAFs, with the exception of TRAF4, are generally considered to be cytoplasmic proteins, and their function has mostly been studied with respect to interactions that take place in the cytoplasm of cells. However, TRAF3, but not other TRAFs, associates with p62 nucleoporin in a HEK293T epithelial cell overexpression system (82). Recently B cell TRAF3 was revealed as a resident nuclear protein; in this role, it functions to restrain transcriptional activation mediated by cyclic AMP response element binding protein (CREB), with which it displays preferentially nuclear association (83). In TRAF3-deficient B cells, there are increased levels of nuclear CREB. This appears to be because in WT B cells, TRAF3 recruits TRAF2 to the nucleus to mediate K48-linked polyubiquitination of CREB, followed by CREB degradation (83). As a result, the pro-survival CREB target Mcl-1 is increased at both mRNA and protein levels in TRAF3-deficient B cells (83). This Mcl-1 increase is consistent with their enhanced homeostatic survival (29). TRAF3 was also shown to contain a functional nuclear localization sequence (NLS) in the TRAFC domain; transfection of TRAF3-deficient B cells with TRAF3 containing a mutated NLS allowed the generation of cells with TRAF3 located predominantly in the cytoplasm. These B cells also show increased CREB-regulated transcription, while cells transfected with WT TRAF3, present both in the cytoplasm and nucleus, do not (83). Although our focus in this review is B cells, it is worth noting that nuclear localization of TRAF3 has also been identified in endothelial cells and neurons (84–86) and TRAF3 forms a transcriptional complex with TRAF2, phospho-RNA Polymerase II and p65/RelA in Neuro2a cells activated through CD40 (84). CD40 is expressed on antigen-presenting cells, including B cells, so this result is of particular interest. It will be interesting and informative to identify additional nuclear binding partners and functions for TRAF3 in both B cells and other cell types.

## TRAF3 and B cell metabolism

B lymphocytes are seldom studied as a key cell type regulating mammalian metabolism, so it is unsurprising that the impact of TRAF3 upon B cell metabolism is to date an understudied topic—but one with intriguing initial findings, as described below. In recent years, manipulation of amounts of TRAF3 in different cell types revealed TRAF3-mediated regulation of a number of metabolic events, leading to striking *in vivo* effects in animal models. In several mouse models of obesity, genetic deletion of *Traf3* in macrophages and neutrophils alleviates a number of hallmarks of obesity-related inflammation. These include insulin resistance, hyperglycemia, glucose intolerance and hepatic steatosis, as well as liver and adipose tissue production of inflammatory cytokines. Conversely, the amounts of these cytokines are increased in the liver and adipose tissue of lean mice (87). A similar pattern was seen for hepatocyte TRAF3, which is low in fasted mice, but increased when glucose levels are elevated by various metabolic manipulations. As with mice lacking myeloid TRAF3, deletion of hepatocyte TRAF3 reduces metabolic abnormalities seen in obese mouse models, while overexpression of TRAF3 in the liver induces metabolic abnormalities and suppresses insulin signaling (88).

Another non-immune cell type in which TRAF3 is reported to regulate metabolic pathways is neural stem cells. These cells are of interest because maternal diabetes is associated with increased neural tube defects, in which caspase-induced apoptosis is thought to play an important role. *Traf3* is a target of microRNA-322; in a mouse model of diabetes, maternal disease and high glucose decreases microRNA while *Traf3* expression and caspase-mediated apoptosis of neural stem cells is increased. Use of a microRNA-322 mimic or inhibition of *Traf3* expression blocks both these effects (89).

In B lymphocytes, TRAF3 functions to restrain rather than promote glucose metabolism, emphasizing the context-dependent nature of TRAF3 functions. TRAF3-deficient B cells express elevated levels of the glucose transporter Glut1 and the glycolytic enzyme Hexokinase 2 (HXK2) (90). This is relevant to the frequent loss of TRAF3 function in B cell malignancies (see below), and also with the well-discussed roles of HXK2, Glut1, and glucose metabolism in many types of cancers [reviewed in (91, 92)]. Consistent with their overexpression of Glut1 and HXK2, TRAF3^−/−^ B cells show enhanced glucose uptake both *in vitro* and *in vivo* (90), as well as increased anaerobic glycolysis and oxidative phosphorylation, without changes in reactive oxygen species or mitochondrial mass (90). Interestingly, although the enhanced viability of TRAF3^−/−^ B cells is not abrogated by deficiency in the TRAF3-regulated kinase NIK (48), TRAF3-controlled Glut1 levels and glucose uptake return to normal if TRAF3-deficient B cells are also rendered NIK-deficient (90).

Increased glucose utilization by TRAF3^−/−^ B cells does contribute to their increased homeostatic survival, however, and renders them more sensitive than TRAF3^+/+^ B cells to death induced by glucose deprivation (90). Human B cell lymphoma (BCL) cell lines also display an inverse correlation between Glut1 and TRAF3 expression, and cell lines with relatively lower TRAF3 expressed show increased sensitivity to glucose deprivation (90). As described above, B cell TRAF3 is a resident nuclear protein that induces degradation of CREB, hence inhibiting transcription of CREB-promoted survival proteins, such as Mcl-1 (83). In the absence of glucose, this upregulation of Mcl-1 induced by TRAF3 deficiency is abrogated (90). Thus, B cell TRAF3 as a metabolic reprogramming protein has particular relevance for B cell malignancies, discussed in more detail below.

## TRAF3-mediated regulation of Pim2 and c-Myc

As described above, TRAF3 limits B cell survival by altering the stability of key kinases and transcription factors through post-translational modification. Recently, it was found that TRAF3 also inhibits expression of the transcriptionally-regulated pro-survival kinase proviral insertion in murine lymphoma 2 (Pim2) (93, 94), a kinase required for the cytokine BAFF to promote B cell survival (95). Interestingly, Pim2 is overexpressed in multiple human cancer types (96, 97), including the B cell malignancies most frequently associated with *TRAF3* deficiency—multiple myeloma (MM) and B cell lymphoma (BCL) (98, 99). TRAF3-deficient primary B cells, as well as MM and BCL cell lines, display an inverse relationship between TRAF3 and Pim2 protein levels (93, 94). TRAF3-deficient B cells have enhanced phosphorylation of the Pim2 targets Bcl-2-associated agonist of cell death (BAD), phospho-ribosomal protein S6 kinase beta-1 (p70S6K), and eukaryotic translation initiation factor 4E-binding protein 1 (4E-BP1) (93, 94), which abrogates BAD-induced apoptosis (100), and relieves p70S6K and 4E-BP1 -mediated translational repression (101). Transcription-independent elevation of the proto-oncogenic protein c-Myc is also observed in TRAF3-deficient B cells. This increase is associated with a striking decrease in K48-linked poly-ubiquitination of c-Myc in these cells. Interestingly, siRNA to Pim2 also reduces these increased levels of c-Myc (93, 94). Consistent with this relationship, combined pharmacological targeting of c-Myc and Pim2 proved significantly more effective in promoting B cell apoptosis than either alone, and TRAF3-deficient B cells are especially sensitive to these drugs (93, 94). The reported cardiac toxicity of single-agent Pim inhibition has limited its clinical utility (102); combining Pim inhbitors at lower doses with c-Myc inhibition could potentially address this problem, particularly in TRAF3-deficient B cell malignancies.

## TRAF3 and B cell malignancies

Human *TRAF3* mutations associated with B cell malignancies were first described in the plasma cell cancer, MM; the nature of such mutations is such that they are expected to be loss-of-function alternations (103, 104). It is now reported that *TRAF3* is one of the top ten mutated genes found in ~ 65% of cases of human MM (105); overall, 15–20% of human MM display *TRAF3* mutations. *TRAF3* gene mutations or loss have also been reported in Hodgkin's disease (106), Waldenstrom's macroglobulinemia (107) and various types of BCL (108). The percentage of BCL with *TRAF3* mutations or deletions varies among studies, but up to 15% of human Diffuse Large BCL (DLBCL) examined show *TRAF3* genetic changes (109, 110, 111). Monoallelic deletions of *TRAF3* are the most common finding (107, 109, 110) and these deletions tend to be large, with mapped deletions from 13 human DLBCL showing a minimum common region of about 600 kb (110). A recent paper analyzing DLBCL by genetic subtype based on clusters of genetic changes showed that *TRAF3* gene loss is frequently, although not exclusively, associated with mutations in *BCL6* and *Notch2* (BN2 subtype) and *Notch1* (N1 subtype) of DLBCL (111). Non-Hodgkin lymphomas are the most common cancers in pet dogs (110). An examination of 84 such canine BCL showed an unexpectedly high 44% bearing *Traf3* mutations, with 30% of these being somatic changes and 14% single-allele germline mutations (110).

As discussed above and elsewhere in this issue, TRAF3 protein plays an important role in the regulation of B cell NF-κB2 activity. Thus, it is not surprising that *TRAF3* gene deletions and mutations in human B cell cancers correlate with an increased NF-κB transcriptional signature (104, 107, 112). However, enforced expression of the NF-κB2-activating kinase NIK in mouse germinal center B cells does not lead to rapid development of BCL, unless *Bcl6* over-expression is also enforced (109). Consistent with this finding, mice engineered to lack TRAF3 in their B cells (B-*Traf3*^−/−^), described in earlier sections, do not develop spontaneous BCL until ~ 8 months of age (113).

In B-*Traf3*^−/−^ mice, B cells exhibit an abnormally long lifespan, resulting in accumulation of B cells in various tissues (29). In these mice, NF-κB2 activity is constitutively elevated in B cells (29). However, as mentioned earlier, the enhanced B cell lifespan is due not only to NF-κB2 activity, but also to other factors, including enhanced CREB activity, with the latter resulting in increased expression of the pro-survival protein Mcl-1 (83). These mice, with *Traf3* deleted in the transitional stage of B cell development using CD19^Cre^ (29), are particularly prone to develop high grade marginal zone BCL with high penetrance (113), consistent with their especially high accumulation of marginal zone B cells (29). These BCL are monoclonal or oligoclonal (113), indicating that absence of *Traf3* is not in itself sufficient to cause BCL, but the enhanced viability such loss confers upon B cells is likely to potentiate their extended survival in the presence of additional mutations.

While *TRAF3* genetic loss is associated with B cell malignancies, this is not the only mechanism by which a B cell can become TRAF3 protein-deficient, with the tumor-predisposing consequences discussed above. Our laboratory recently reported the results of TRAF3 protein staining of several 100 human DLBCL samples, which revealed that more than 30% of these BCL had low to undetectable TRAF3 protein expression (114). It was previously demonstrated that the EBV transforming protein LMP1 binds TRAF3 with considerably enhanced affinity, compared to the normal cellular receptor that it mimics, CD40 (21, 23). Thus, we examined whether B cell expression of LMP1 is associated with sequestration of TRAF3 in the plasma membrane, resulting in decreased availability of TRAF3 to downregulate various pro-survival signaling pathways discussed above; this was found to be the case (114). It is also well-documented that signaling to B cells via CD40 or BAFFR leads to poly-ubiquitination and degradation of TRAF3 [reviewed in (33)]. A decrease in TRAF3 protein expression in B cell tumors without detectable *TRAF3* gene changes could thus also be the result of chronic signaling through CD40 or BAFFR, or other receptors that activate TRAF3 degradation. This is an intriguing possibility for future investigation. Thus, many more B cell cancers may be impacted by the biologic pro-survival impact of TRAF3 deficiency than even the significant number impacted by *TRAF3* gene loss.

## Conclusions

Although prior reviews have discussed TRAF3 functions in general [e.g., see (115, 116)], the underlying assumption has been that functions defined in one cell type or model system apply to all cell types and TRAF3-binding receptors. However, as discussed in the present review, while some roles for TRAF3 overlap between cell types, there are many and varied biological roles for this pleiotropic signaling protein that are quite context-specific. TRAF3 is particularly important in regulating B lymphocytes, due to its B-cell-specific role in restraining homeostatic survival. As discussed above, TRAF3 also has many additional roles in B cell biology (Figure 1), many of which contribute to its increasingly-appreciated function as a B cell tumor suppressor. Our discussions above also highlight many interesting knowledge gaps that remain to be filled in understanding B cell TRAF3.

## Author contributions

GB, BH, and LS all contributed to planning, writing, and editing this review. GB was responsible for final organization.

### Conflict of interest statement

The authors declare that the research was conducted in the absence of any commercial or financial relationships that could be construed as a potential conflict of interest.

## Abbreviations

4E-BP1: eukaryotic translation initiation factor 4E-binding protein 1
APRIL: a proliferation-inducing ligand
BAD: Bcl-2-associated agonist of cell death
BAFF: B cell activating factor
BCL: B cell lymphoma
BCMA: B cell maturation antigen
B-*Traf3*^−/−^: mice engineered to lack TRAF3 specifically in B cells
CREB: cyclic AMP responsive element binding protein
DLBCL: diffuse large BCL
EBV: Epstein-Barr virus
ERK: extracellular-regulated kinase
Glut1: glucose transporter 1
HXK2: hexokinase 2
cIAP: cellular inhibitor of apoptosis
IκB: inhibitor of kappa B
IKK: IκB kinase
IL: interleukin
IFN: interferon
IRF: IFN response factor
DC: dendritic cells
Jak: Janus kinase
JNK: c-jun kinase
LMP1: latent membrane protein 1
MAPK: mitogen-activated protein kinase
MAP3K: MAP kinase kinase kinase
MM: multiple myeloma
NF-κB: nuclear factor of kappa B
NIK: NF-κB-inducible kinase
NLS: nuclear localization signal
p70S6K: phospho-ribosomal protein S6 kinase beta-1
PI3K: phosphatidyl inosital 3-kinase
Pim: proviral insertion in murine lymphoma
PTP: protein tyrosine phosphatase
siRNA: small interfering RNA
Stat: signal transducer and activator of transcription
TACI: transmembrane and CAML interactor
TCPTP: T cell protein tyrosine phosphatase
TLR: toll-like receptor
TNFR: tumor necrosis factor receptor
TNFRSF: TNFR superfamily
TRAF: TNFR-associated factor.
